# Multiscale and Multimaterial Fabrication: The Challenge Ahead

**DOI:** 10.3390/mi7100178

**Published:** 2016-10-01

**Authors:** Nam-Trung Nguyen

**Affiliations:** Editor-in-Chief of *Micromachines*, Queensland Micro- and Nanotechnology Centre, Griffith University, 170 Kessels Road, Brisbane, Queensland 4111, Australia; nam-trung.nguyen@griffith.edu.au; Tel.: +61-7-3735-3921; Fax: +61-7-3735-8021

In the editorial published in March 2016, I mentioned that one of the aims of *Micromachines* is to cover topics and technologies beyond silicon-based microsystems and microdevices [1]. In fact, broadening application areas of micromachines beyond the traditional silicon-based sensors and actuators brings new challenges to the development of microfabrication technology. Conventional Micro Electro Mechanical Systems (MEMS) and Nano Electro Mechanical Systems (NEMS) are in the size range of micrometers and can be fabricated in batches using well-established photolithography and etching processes. Even with the integration of microelectronics, devices are often self-contained and easily packaged. Novel applications such as those in the areas of microfluidics and lab-on-a-chip (LOC) require device features in a size scale that differs with orders of magnitude. The mismatch in feature size and the multiscale nature of these devices bring new challenges to the device design, as well as the development of economic and large-scale fabrication processes. Imagine that one has to fabricate centimeter-large devices with precise features in the submicron scale. The large footprint and the precision required would make even batch fabrication on silicon substrate economically prohibitive. Thus, the challenge would be the integration of device components of different size scales, made of, different materials and by different fabrication techniques into the final product. The hybrid integration approach of traditional packaging provides a solution for this challenge. Recent papers published in *Micromachines* illustrate the diverse solutions for multiscale and multimaterial challenges posed.

Esashi and Tanaka [2] reported a stacked integration method as a wafer-level packaging solution for large scale integration (LSI) electronics and MEMS devices. The stacking method requires good wafer-to-wafer alignment and an adhesive layer to transfer a thin silicon device layer to an existing LSI wafer. Electrical contacts could be made in the same fabrication process for MEMS devices or as the last fabrication step. Another method transferred a different device material (lithiumniobate) to the LSI silicon wafer through gold bumps. Selective transfer can be achieved using a laser beam to burn out the polymeric adhesive holding the MEMS device. These transfer methods are suitable for devices made of different material systems but of the same size scale.

Kim et al. [3] demonstrated a solution for the multiscale challenge in the fabrication of a LOC device for axon growth. While the guiding channel for the axons needs to be a few micrometers deep, the reservoir for the culture medium could be as large as one centimeter, and the compartment for the neuronal soma could be at the millimeter scale. The authors reported a fabrication approach called the micro–macro hybrid soft lithography master fabrication method and a model of the device was made in poly(methyl methacrylate) (PMMA). While the millimeter scale features were fabricated with precision milling, the micrometer scale features were transferred from a micromachined silicon master through hot embossing. A polydimethylsiloxane (PDMS) master mold was then cast from the PMMA model. The master mold had both micrometer scale and millimeter scale features and was used for casting the actual device in PDMS. The elastic PDMS master enables the fabrication of multiple through holes by pressing against a solid glass slide. Thus, not only multiscale fabrication, but also large-scale fabrication of microfluidic access holes were realized using this approach. 

PDMS has not only been used as master and device material, but also as a convenient medium for the integration of different materials in a multimaterial device. Ma et al. [4] used PDMS to package grating structures made of thick-film resist SU-8 and a conventional glass waveguide to make long-period fiber grating for strain measurement. The combination of rather “exotic” materials could provide a novel solution to long standing problems. Chen et al. [5] used a thermo-reversible gel to support a high-aspect ratio structure, which is prone to collapse due to the capillary force. For subsequent processes, such as electroplating, the gel can simply be dissolved by increasing the temperature of the liquid bath.

Examples from the last three issues of *Micromachines* (July–September 2016) indicated that the research community has already provided some initial solutions to the challenges of multiscale/multimaterial fabrication. It seems that pattern transfer through molding or hot embossing could be practical and economic approaches. The integration of more complex functional devices made from different technologies could help in the ongoing development of wafer-level packaging. As current solutions are rather ad-hoc and specific to certain applications, more research and development are needed in the near future to provide a more systematic solution for the overall challenges of multiscale/multimaterial fabrication.
